# Supplementary material for: Multianalyte serology in home-sampled blood enables an unbiased assessment of the immune response against SARS-CoV-2
Source: Nat Commun. 2021 Jun 17;12:3695. doi: 10.1038/s41467-021-23893-4 (PMC8211676; doi:10.1038/s41467-021-23893-4)
Supplement: Supplementary file 1 — Supplementary Information [file 41467_2021_23893_MOESM1_ESM.pdf]

Supplementary

Multianalyte serology in home-sampled blood enables an unbiased assessment of the immune response against SARS-CoV-2

Authors: Niclas Roxhed[1,2,*], Annika Bendes[3,§], Matilda Dale[3,§], Cecilia Mattsson[3,§], Leo Hanke[4], Tea Dodig-Crnković[3], Murray Christian[4], Birthe Meineke[5,6], Simon Elsässer[5,6], Juni Andréll[7], Sebastian Havervall[8], Charlotte Thålin[8], Carina Eklund[9], Joakim Dillner[9], Olof Beck[10], Cecilia E Thomas[3], Gerald McInerney[4], Mun-Gwan Hong[3], Ben Murrell[4], Claudia Fredolini[3] and Jochen M Schwenk[3,*]

Supplementary Table of Content:

Supplementary Notes: 3
1) Assay development 3
2) Evaluate carry-over effects in consecutive sample extraction 4
3) Determine assay precision 5
4) Assessment of detectability 5
5) Longitudinal resampling analysis 6
5) Pilot study for method validation 6

Supplementary Figures: 9
Fig. S1. Detailed description of translational serology workflow and assays. 9
Fig. S2. Carry-over assessment. 10
Fig. S3. Limit of detection assessment. 11
Fig. S4. Longitudinal resampling analysis. 12
Fig. S5. Comparison of sample types and serology assays. 13
Fig. S6. Serology profiles from ELISA and multiplexed SBA assays in plasma and DBS. 14
Fig. S7. Direct comparison of antibody levels sample types and serology assays 16
Fig. S8. Global comparison of sample types and serology assays. 18
Fig. S9A. IgG serology with study set 1. 19
Fig. S9B. IgM serology with study set 1. 19
Fig. S9C. IgG serology with study set 2 20
Fig. S9D. IgM serology from study set 2. 21
Fig. S10A. PCA analyses of study set 1 22
Fig. S10B. PCA analyses of study set 2. 22
Fig. S11A. IgG profiles of UMAP-selected seropositive and seronegative individuals. 23
Fig. S11B. IgM profiles of UMAP-selected seropositive and seronegative individuals. 24
Fig. S12A. Overlap in seropositive samples classified for IgG and IgM per study set. 25
Fig. S12B. Prevalence of IgG against SARS-CoV-2 proteins in study set 1. 26
Fig. S12C. Prevalence of IgM against SARS-CoV-2 proteins in study set 1. 26
Fig. S12D Prevalence of IgG against SARS-CoV-2 proteins in study set 2. 27
Fig. S12E Prevalence of IgM against SARS-CoV-2 proteins in study set 2 27

Supplementary Tables: 28
Table S1. Inter- and Intra-day variability. 28
Table S2. Demographic characteristics of the pilot study. 29
Table S3. Demographics of two study sets from random population sampling 30
Table S4. Seroprevalence per antibody isotype and SARS-CoV-2 protein. 31
Table S5. Demographics of subjects in UMAP group. 32
Table S6. Demographics of subjects dual-S group (including all samples) 33
Table S7. Comparisons of dual-S and UMAP group. 34

Supplementary Notes:

1) Assay development

All dried blood cards utilized in the project were subjected to heat-inactivation prior to any handling. To begin with, EDTA plasma samples were applied onto the discs to adjust the protocol in terms of elution volumes, buffer compositions and incubation times (data not shown). The elution of the proteins from the cards was performed in 100 µl of detergent-containing buffer over 60 min and gentle shaking at room temperature. Each disc adsorbed a small volume of the supplied buffer so that a volume of ~ 70 µl could be harvested as supernatant for further analyses. The protein concentration of the eluates was determined to be about 5 mg/ml (± 0.5 mg/ml). Subjecting the disc to another round of elution reduced the protein concentration to about 2 mg/ml (± 0.4 mg/ml). For the multiplexed assays, 16 µl were used at a 1/2.5 dilution. This enabled 3-4 serology assays from one disc or 6-8 assays per card in case both discs were properly filled with blood.

The composition of the elution buffer was based on PBS containing 0.05% Tween20 (PBST). To check if the addition of a protease inhibitor cocktail (PIC) and protein (0.5% BSA) would have any effect, the consistency of IgG reactivity levels for SPK_01, RBD_01, RBD_02, NCP_01, EBN_01, SPS_01 as well as beads carrying anti-human IgG or no protein (bare beads) were tested. All elution buffers provided high correlations between data obtained from the two discs (rho = 0.99). The results using the buffer containing PIC were though more consistent in intensity values (slope [PBST/PIC] = 0.99) compared to the other buffers (slope [PBST] = 0.85; slope [PBST/BSA] = 0.89). Elution was therefore conducted using PBST with PIC but without BSA.

The investigation studied the antibodies as well as proteins in DBS eluates from discs having dried blood from a volume of 10 µl. All sample processing steps were compatible with well-established immunoassay protocols for serum or plasma samples. Since whole blood consists

of about 50% cell free fluid, we used this approximation to calculate the dilution factors for our assays. While further optimization of the sample preparation is possible, the addition of Tween20 and protease inhibitor cocktails were sufficient to obtain 70 µl of eluate that contained 350 µg of protein. This eluate contained about 70% of the theoretical 500 µg of plasma proteins in 10 µl of blood from the discs. The main utility of the protocol was to determine circulating levels of IgG and IgM, which are all abundant and stable blood proteins.

2) Evaluate carry-over effects in consecutive sample extraction

To further streamline the workflow, a semi-automated punching device was utilized that ejected one disc per card into a designated well of a 96-well plate. Consequently, the punching device was used to transfer blood-loaded discs in a semi-automated manner. Since sequential ejection of cards might bear a risk of protein carry-over, the amount of proteins brought onto a subsequently ejected disc was evaluated. Three discs were filled with venous blood from a PCR-positive donor. The discs were ejected followed by three empty discs. All discs were then subjected to protein-elution and analysed for their IgG reactivity levels towards several SARS-CoV-2 and antigen from the prevalent Epstein–Barr virus were determined. Since the exact amounts of antigen-specific antibodies were unknown, a relative carry-over was determined. The data was normalized setting the blood-loaded disc as 100% and measurements in pure assay buffer as 0% to account for different background levels and ranges of fluorescence signal intensities for each antigen. Log-transformed data from the triplicated measurements was used for the analysis to avoid a difference in judging changes occurring at higher (MFI > 1000 [AU]) or lower (MFI < 100 [AU]) intensity levels. As shown in **Fig. S2.**, the relative carry-over remained < 15% for the first empty disc for the different antigen. Only the detection of IgG on consecutive discs was elevated. Nonetheless, the effects of relative carry-over from a PCR-positive control sample onto empty discs was found to be acceptable.

3) Determine assay precision

To further characterize the developed procedure, the repeatability (intra-day variability), and reproducibility (inter-day variability) aspects were evaluated. For this purpose, four DBS discs loaded with blood from two donors, one being PCR-positive and the other being seronegative: The DBS were processed and analyzed in quadruplicates over four days. The coefficients of variation (CV) were determined from the standard deviations (SD) of log-transformed data, obtained as median fluorescence intensity (MFI) values, using the formula:

Equation 1: $$\mathrm{CV} = \sqrt[2]{\mathrm{e}^{SD_{\mathrm{ln}}^{2}} - 1}$$

As summarized in **Table S1.**, the CVs remained below 30%, and as expected were slightly higher between days (18%) than within days (13%).

4) Assessment of detectability

The performance of the multiplexed serology assay was further studied by assessing the detectability with limits of blank (LOB) and limit of detection (LOD). Here, the DBS eluates from a PCR- and seropositive sample was diluted into DBS eluates pooled from seronegative samples. The following foreign were used, where 'blank' referred to sample containing only assay buffer and 'negative' referred to a sample containing the eluates from the seronegative participants:

Equation 2: $$\mathrm{LOB} = \overline{MFI}_{\mathrm{blank}} + 1.645 \cdot SD_{\mathrm{blank}}$$

Equation 3: $$\mathrm{LOD} = \overline{MFI}_{\mathrm{blank}} + 1.645 \cdot SD_{\mathrm{negative}}$$

A serial dilution series was prepared in steps of three-fold dilutions (**Fig. S3**). For most antigens, the applied dilution was close to an optimal dilution with little neglectable hook-effects. The LOBs were about 10% above the levels in the blank, while the LODs were about 30% above the levels determine in the blank.

5) Longitudinal resampling analysis

To investigate the technical performance related to repeated sampling and assess how IgG and IgM levels against the several different SARS-CoV-2 antigens changed over time, a single PCR-positive donor collected blood on the DBS cards two weeks after a self-reported onset of COVID-19 symptoms. Blood was collected at five occasions over two weeks in intervals of about 3-8 days and until 30 days after symptom onset. Samples were analyzed within four weeks post collection with the multiplexed assay. As shown in **Fig. S4.**, levels of IgG antibodies remained stabled for the SARS-CoV-2 proteins, while those of IgM decreased over time.

Linear regression was used to quantify and rank the changes in antibody levels. We found that total levels of circulating levels of IgG (slope = 0.01) and IgM (slope = 0.003) remained unchanged during the sampling period. Combing the slopes from several recombinant RBD, S and N proteins, the IgG levels slightly increased with time (slope = 0.11±0.03). This short-term longitudinal analysis indicated the reproducibility of the workflow. Levels of IgM against the different virus proteins decreased over time (slope = –0.39±0.12), which anti-S (slope = –0.47) and anti-RBD (slope = –0.45) decreasing more than those for anti-N (slope = –0.22). The differences in decline of IgM and stable IgG levels are in line with studies conducted in serum or plasma samples. This further strengthened the suitability of the DBS-based multiplexed serology approach.

5) Pilot study for method validation

To validate the procedure of using DBS eluates, data from commercially available SARS-CoV-2 ELISA assays for the S1 and N proteins were compared with the multiplexed serology. Antibody titers obtained from measuring IgG in blood collected by finger-pricking, venous blood prepared as EDTA plasma, as well as whole blood were evaluated. The samples for this study were collected by trained personnel and obtained from a local health care center. They

reported a high frequency of sick leaves during spring of 2020, hence, a bias for a higher prevalence of antibodies against the virus was expected.

First, antibodies against the S1 and N protein were tested in EDTA plasma and DBS eluates (**Fig. S5A**). In plasma, 50% (25/50) of the samples had IgG antibodies against S1, and concordantly, 48% (24/50) against the N protein. These seropositivity scores overlapped by 92% (23/25), hence two donors deemed positive by only one of the proteins. Next, DBS eluates from a subset of 38 volunteers were prepared and tested at the same theoretical amount as EDTA plasma. As shown in **Fig. S5B-C**, a high degree of concordance was found between DBS and plasma levels of IgG against the S1 and N proteins. Comparing the data obtained from DBS and plasma between the ELISA and respective protein of the SBA revealed a high agreement between the two methods, see Supplementary Data file sheet: "Paired antigens in pilot test". As further shown in **Fig. S6** for the SBA assays, the DBS and plasma data derived from the S, RBD and N protein preparations correlated (rho > 0.9). Minor differences in the classification using DBS or plasma levels were found for samples close to the designated cut-off. Consequently, the very high concordance between antibody levels found in DBS eluates and the corresponding plasma samples confirmed the suitability of DBS workflow for serological analysis.

Further correlation analyses were used to assess the degree of similarity between the ELISA and SBA data within plasma and DBS samples, see Supplementary Data file sheet: "Paired samples in pilot test". For the NCP protein used in the ELISA and the three NCPs used in the SBA assays, there was a supportive similarity for DBS eluates (rho = 0.80±0.05) and plasma (rho = 0.76±0.06). The degree of concordance between S1 ELISA and SBA data for three SPK proteins was even higher in DBS eluates (rho = 0.86±0.005) as well as plasma (rho = 0.89±0.005). The different antigen preparations included in the SBA assay were also compared to obtain insights about the intra-assay consistency of reactivity levels. A high degree of

concordance was determined between the three SPK proteins in DBS eluates (rho = 0.98±0.006) and plasma (rho = 0.99±0), between the four RBD proteins in (DBS: rho = 0.91±0.06; plasma: rho= 0.90±0.06), as well as between three NCP proteins (DBS: rho = 0.86±0.06; plasma: rho = 0.85±0.07). Taken together, these data indicated that the developed procedure performed on par with ELISA assays to identify seropositive and negative samples from plasma as well as DBS eluates, but the SBA's multiplexing capacity provided further details about the diversity of serological profiles for different antigens.

The multiplex SBA workflow was then evaluated for the different sample types and SARS-CoV-2 proteins obtained from different sources were tested. First, two clustering analyses showed no distinct separation between plasma and DBS (**Fig. S7A-D**). There was though a clear separation into two clusters when using ELISA S1 seropositivity as an identifier (**Fig. S7A-B**), and included PCR confirmed convalescent participants grouped withing the ELISA positive cluster. There was only a single subject with borderline ELISA ratios (positive for S1, negative for N) for which all three sample types grouped among the seronegative subjects. Importantly, there was no difference in classification due to the type of the analysed samples (**Fig. S7C-D**). This again strengthened the validity of using DBS for SARS-CoV-2 serology purposes. Concordance between individual antigens and the ELISA data is shown in **Fig. S8** for both sample preparation types. The levels of total IgG and those against Epstein–Barr virus nuclear antigen 1 (EBNA1) did not differ between the seropositivity groups.

Supplementary Figures:

Fig. S1. Detailed description of translational serology workflow and assays.

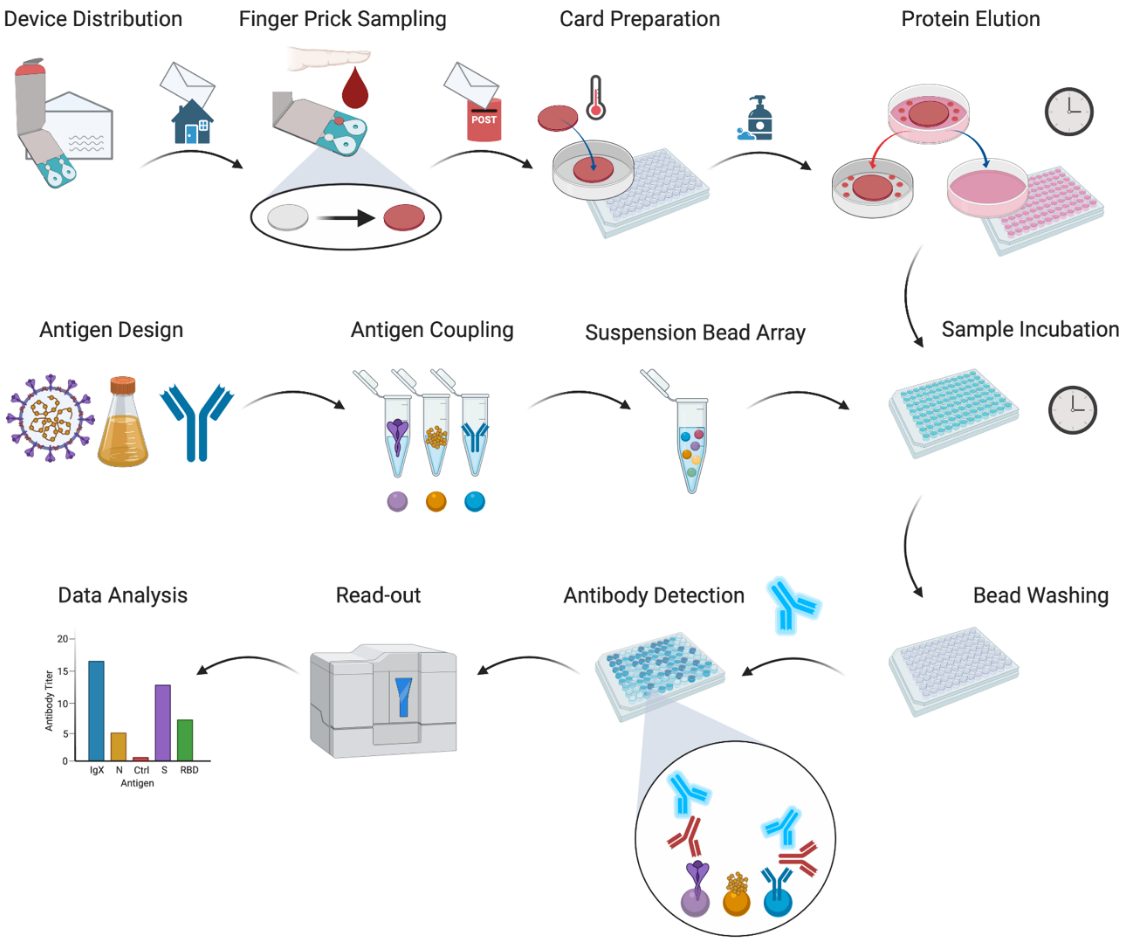

(Top row) Blood collection devices were distributed by postal mail to collect blood samples from finger pricking at home. Cards were then mailed to the laboratory for analysis. There, each card was first heat treated and one disc per card was ejected into microtiter plates. Proteins were carefully eluted from the cards using a detergent containing buffer, and the supernatants were applied for antibody analyses. (Center row) Different viral antigens and other capture reagents were generated or obtained to be immobilized onto designated color-coded beads. A suspension bead array (SBA) was then created by mixing the different protein-coupled beads. The SBA and samples diluted into assay buffer were then combined and incubated. (Bottom row) The magnetic beads were washed to remove unbound sample proteins. Detection antibodies against human IgG or IgM were applied to report the binding of human antibodies against the viral proteins. The beads were then analyzed in a cytometer to obtain median fluorescence intensity values per protein and samples.

Fig. S2. Carry-over assessment.

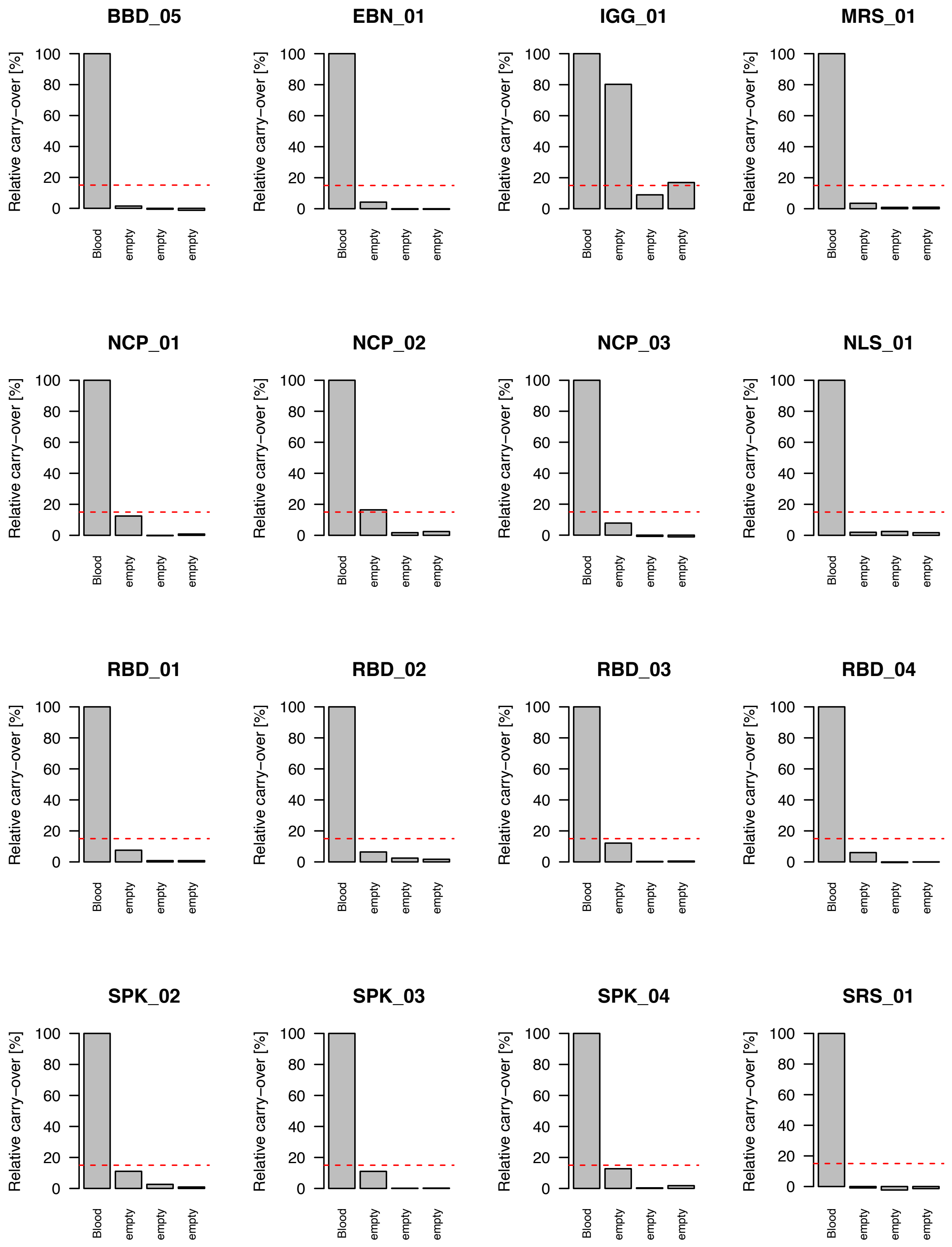

The relative carry-over from a blood-loaded disc onto empty discs was evaluated by sequential punching of cards. The possible transfer of proteins from the blood loaded card was assessed using a relative measure defined by the maximum signal (blood) and minimum (buffer). The red dashed lines indicate a relative carry-over of 15%. Source data are provided as a Source Data file.

Fig. S3. Limit of detection assessment.

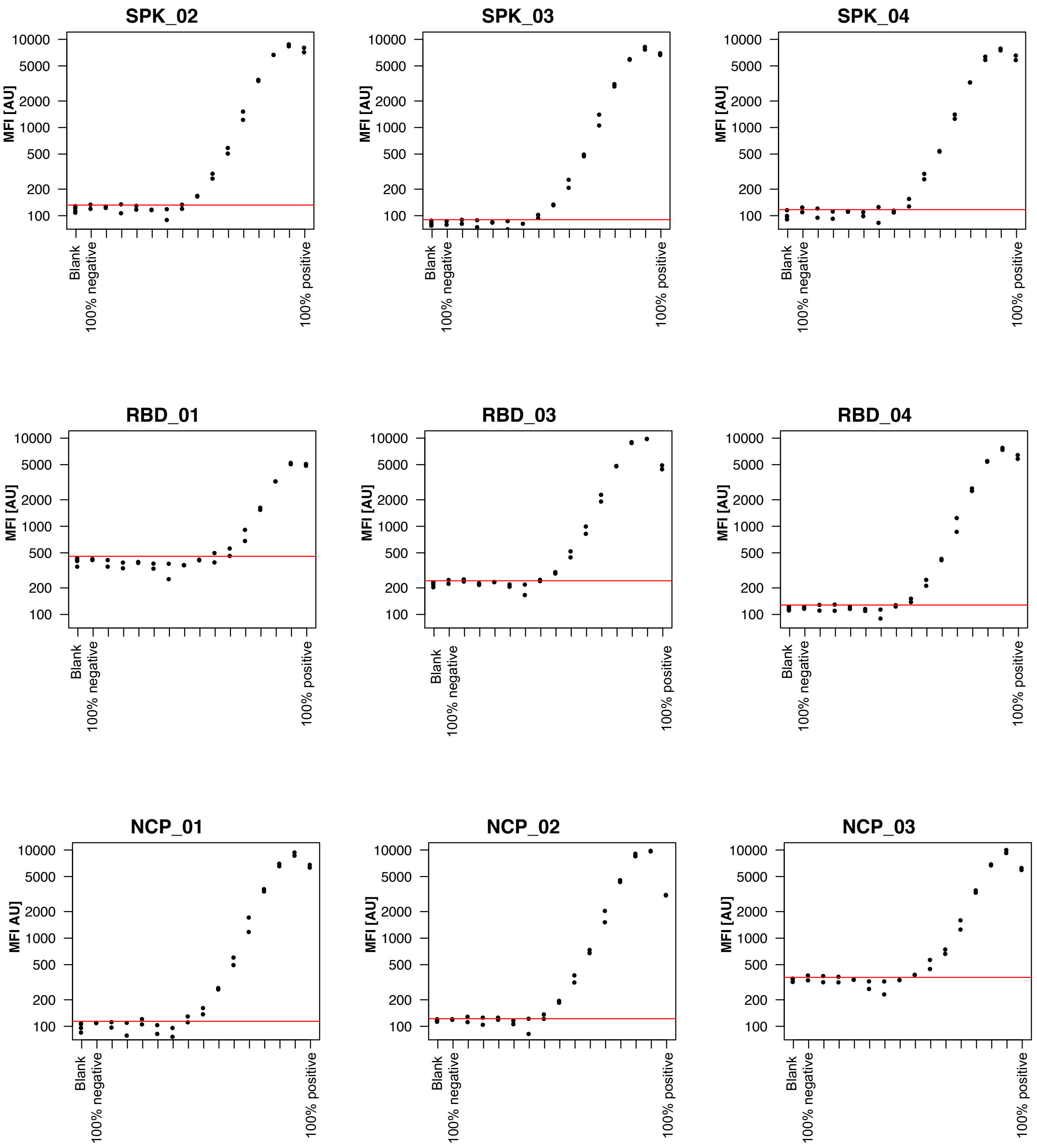

A series of sample-in-sample dilutions were prepared by diluting DBS eluates from a PCR-positive donor into those of seronegative donors. For each antigen, the assay was performed in duplicate. To the left of each plot, a blank sample was used. The proportion of the positive sample increased from 0% (stated as 100% negative) to 100% positive. The latter indicated the concentration used in the described assays. The data represents the detection of IgG levels reported as MFI values (scale of y-axis is log10) and the read lines indicate the limit of detection (LOD). Source data are provided as a Source Data file.

Fig. S4. Longitudinal resampling analysis.

(A)

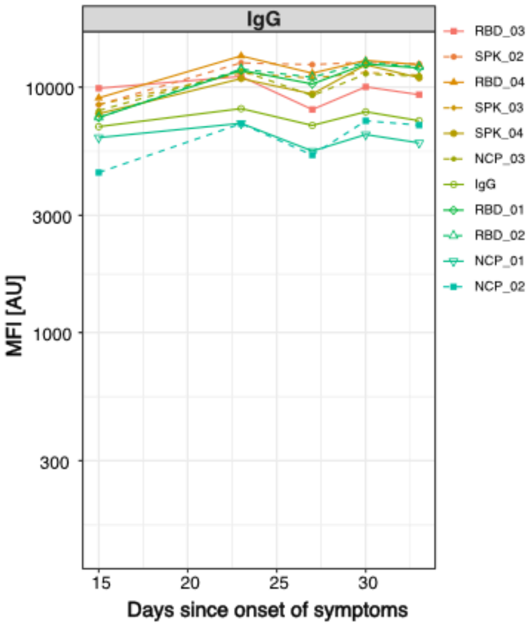

(B)

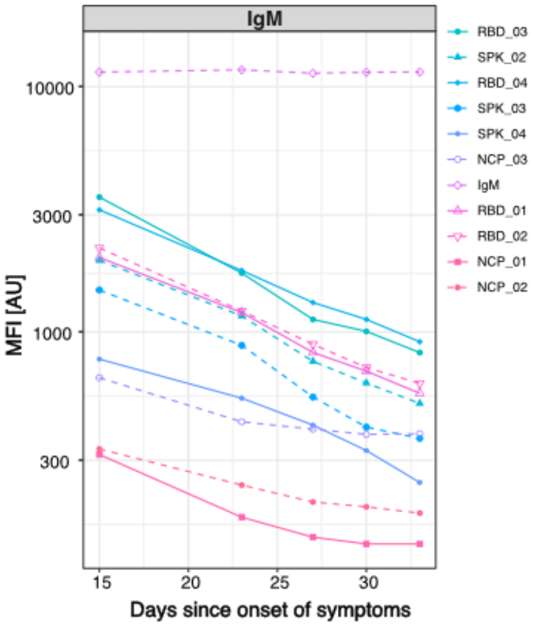

One PCR-positive donor collected blood on DBS cards at five occasions since self-reported onset of symptoms. Eluates from the DBS were analysed for multiple virus proteins for titers of **(A)** IgG and **(B)** IgM. While IgG reactivity levels remained elevated throughout the sampling period, the levels of IgM against SARS-CoV-2 antigens declined over time. The data is presented as MFI values (scale of y-axis is log2) with each antigen presented in a unique colour and symbol. Source data are provided as a Source Data file.

Fig. S5. Comparison of sample types and serology assays.

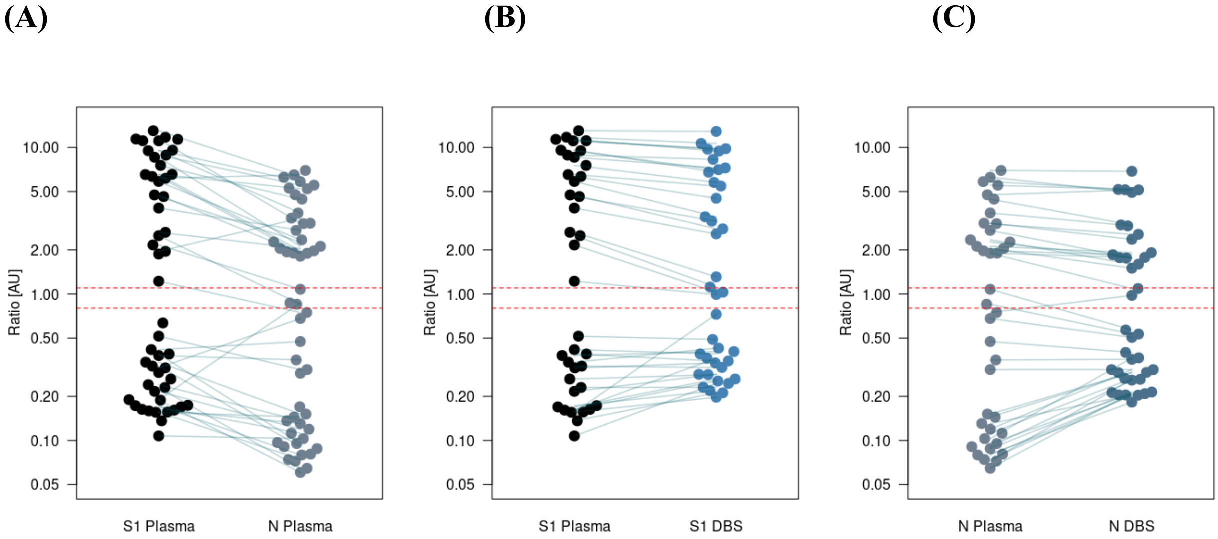

SARS-CoV-2 ELISA with DBS and EDTA plasma samples. (**A**) ELISA were performed on EDTA plasma from 50 subjects to detect IgG antibodies against S1 (black) and the N protein (grey). Ratios between the internal controls are shown with the dotted lines indicating the recommended cut-off levels for seropositivity. Samples between the two red lines are borderline. The correlation between the S1 and N data in plasma was rho = 0.87 **(B)** A comparison between EDTA plasma (black) and DBS eluates (blue) for S1 ELISA with 38 subjects. For S1, the correlation between the plasma data and DBS data was rho = 0.96 **(C)** The same comparison is shown for the N protein in EDTA plasma (grey) and DBS eluates (blue), both showing a good concordance between the sample types. For N, the correlation between the plasma data and DBS data was rho = 0.97. Source data are provided as a Source Data file.

Fig. S6. Serology profiles from ELISA and multiplexed SBA assays in plasma and DBS.

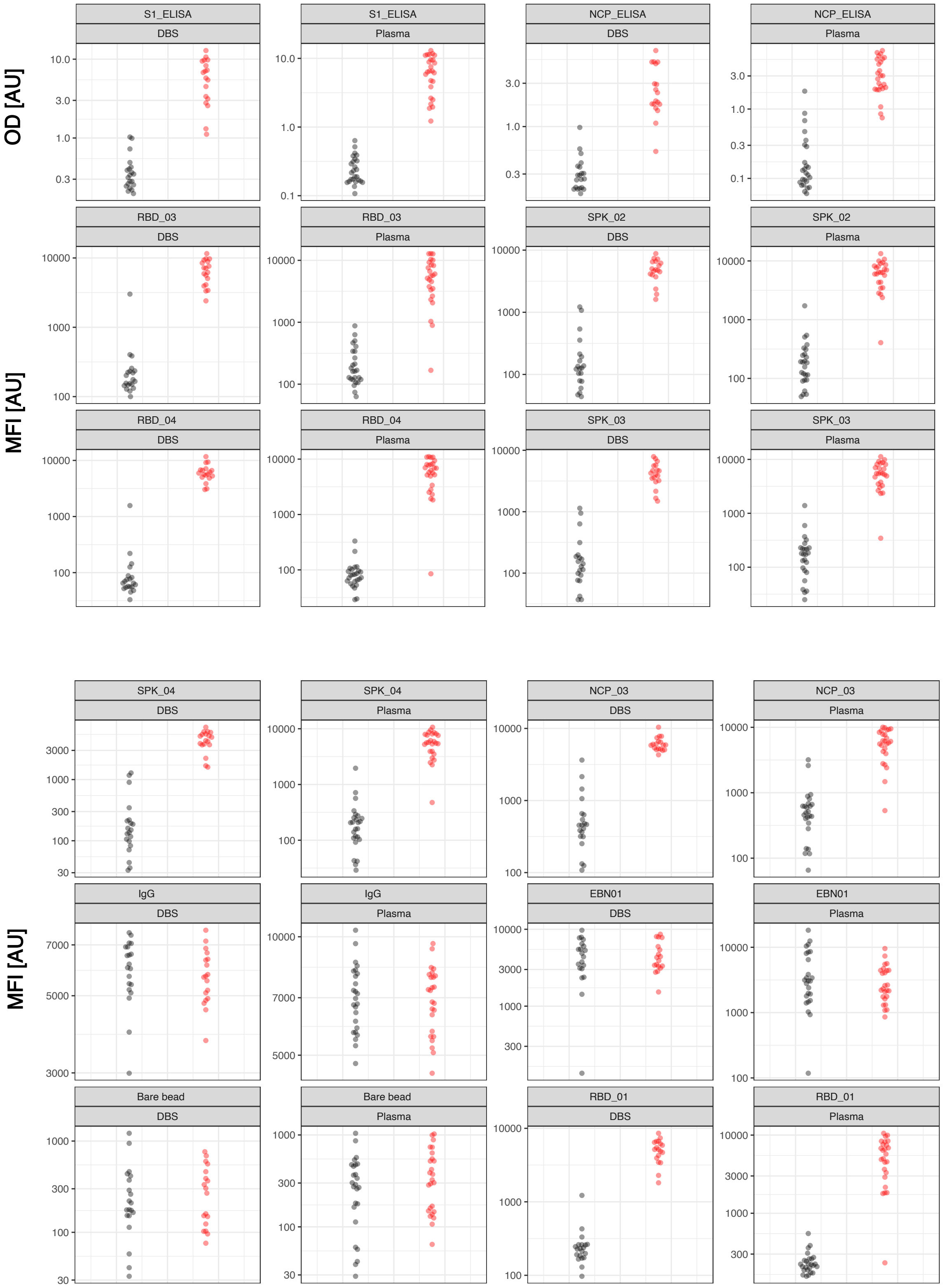

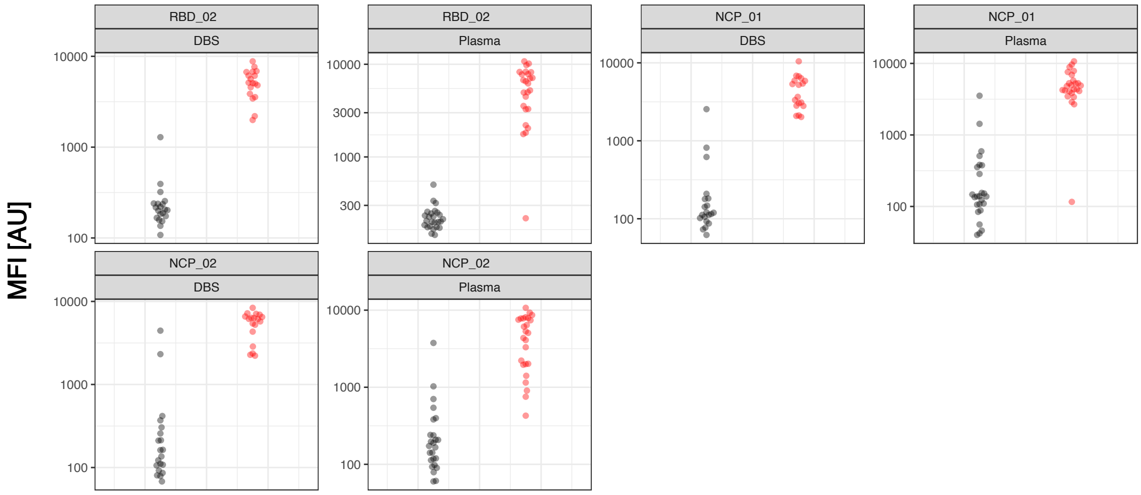

The seropositivity classification of the S1 ELISA was used to determine profiles as negative (black) and positive (red). Each plot shows the outcome of the analysis using plasma and DBS eluates as samples. In the first row, the ELISA data for S1 and N protein are shown. All following plots represent the data of the multiplexed serology assays. In the header of each plot, the antigen name and sample type are shown. MFI values are shown in log10 scale. Source data are provided as a Source Data file.

Fig. S7. Direct comparison of antibody levels sample types and serology assays.

(A)

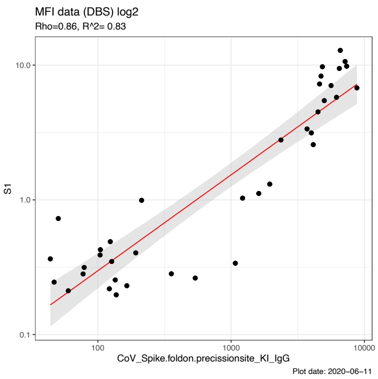

(B)

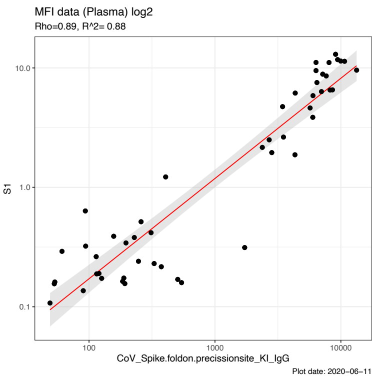

(C)

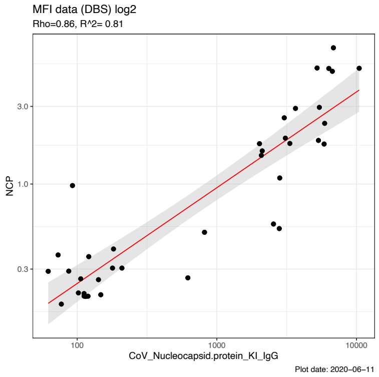

(D)

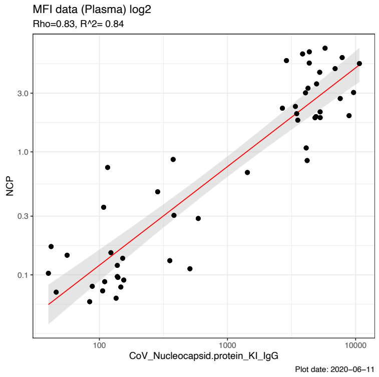

Data from DBS eluates and plasma of ELISA assays for S1 and N (OD, y-axis) were compared with SBA data of **(A-B)** SPK_01 and **(C-D)** NCP_01 (MFI, x-axis, log10 scale). The red lines represent the linear correlations with grey areas being the 95% confidence intervals. Source data are provided as a Source Data file.

(E) **(F)** **(G)**

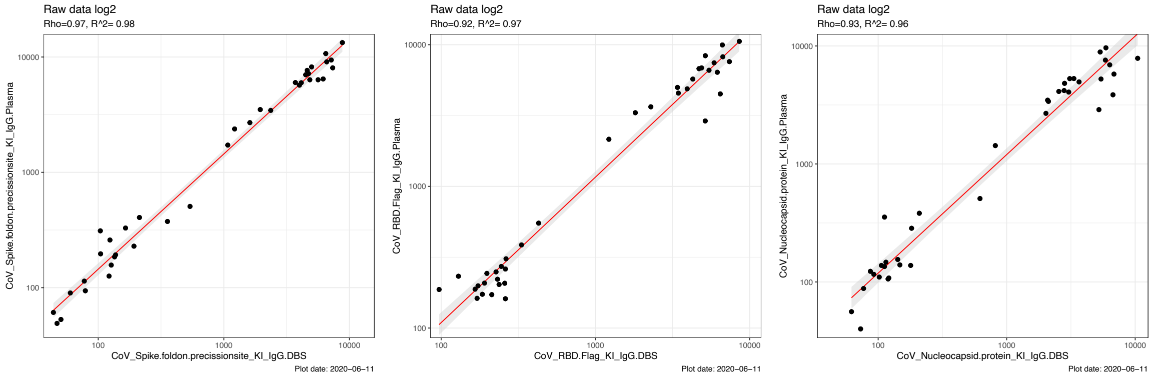

The SBA data obtained in DBS eluates (x-axis, log10 scale) and EDTA plasma (y-axis, log10 scale) of S, RBD and N proteins in 50 donors are represented by **(E)** SPK_01, **(F)** RBD_01 and **(G)** NCP_01, respectively. The red lines represent the linear correlations with grey areas being the 95% confidence intervals. Source data are provided as a Source Data file.

Fig. S8. Global comparison of sample types and serology assays.

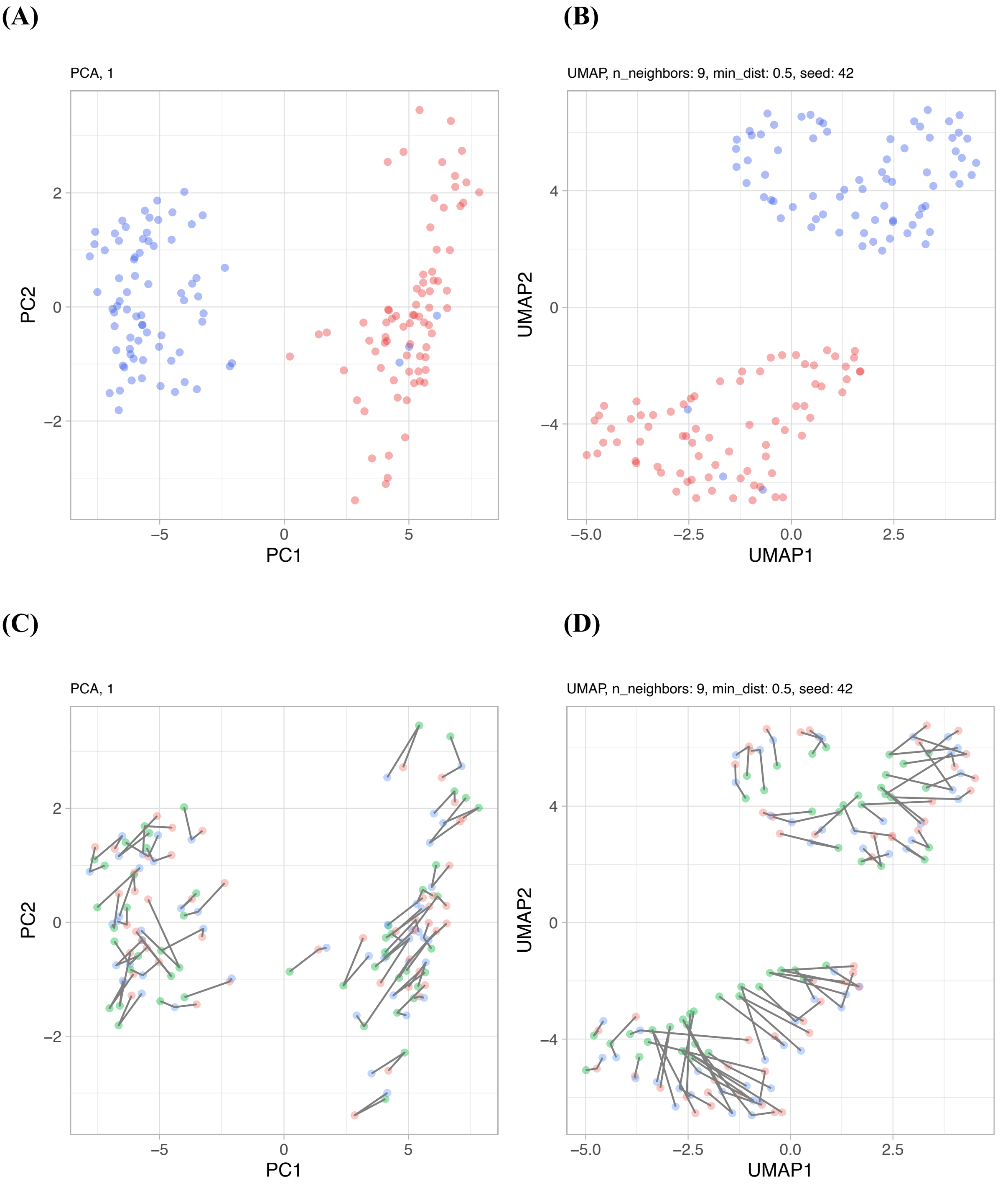

Unsupervised clustering of the multiplexed SBA data was performed using **(A)** PCA and **(B)** UMAP, coloured based on S1 ELISA seropositive (blue) and seronegative (red) subjects. The SBA data separated ELISA S1 seropositive and seronegative samples with one exception. **(C)** PCA and **(D)** UMAP analysis represent the different sample types such as EDTA plasma (green), capillary blood DBS (cDBS, red), and venous blood applied onto DBS cards (vDBS, blue). Samples from the same donors (grey lines) clustered together. Source data are provided as a Source Data file.

Fig. S9A. IgG serology with study set 1.

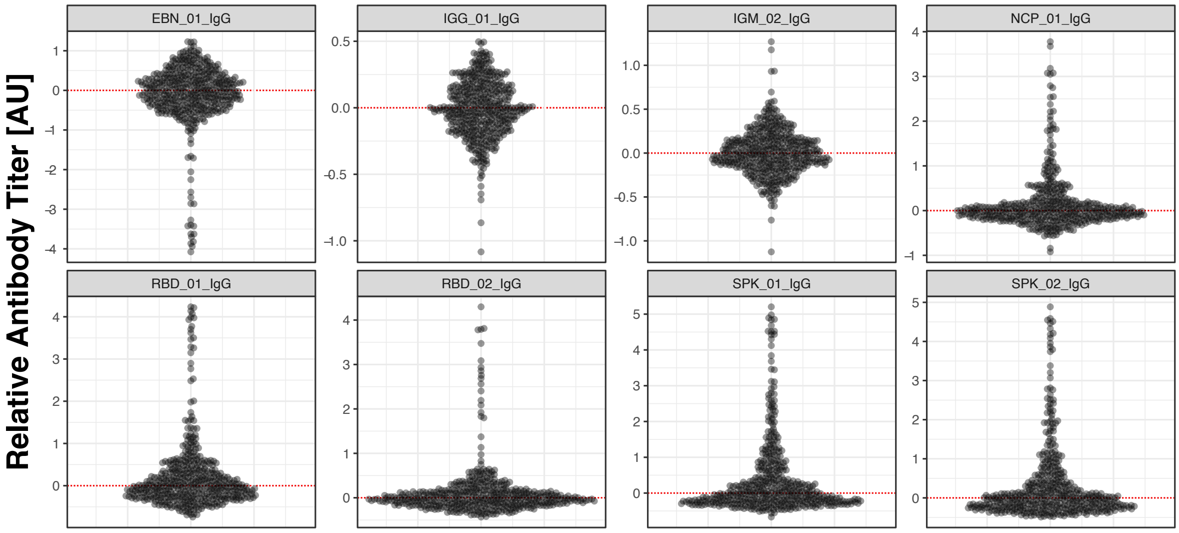

The relative IgG antibody titers detected in DBS are shown per antigen using the acronyms described in Table 1 of the Methods. The data represent the population, negative and positive controls samples. Source data are provided as a Source Data file.

Fig. S9B. IgM serology with study set 1.

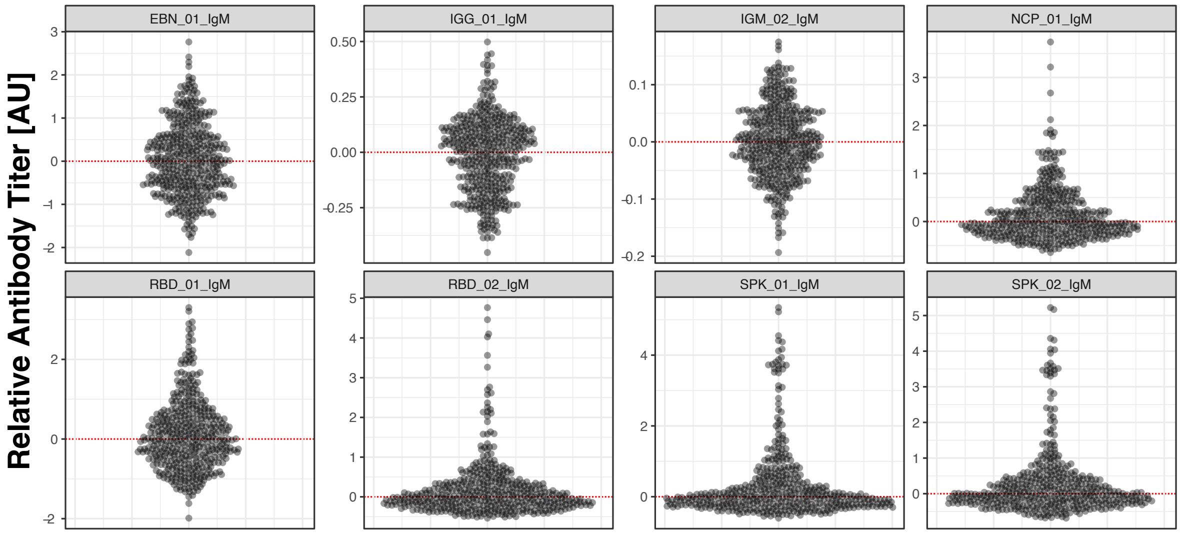

The relative IgM antibody titers detected in DBS are shown per antigen using the acronyms described in Table 1 of the Methods . The data represent the population, negative and positive controls samples. Source data are provided as a Source Data file.

Fig. S9C. IgG serology with study set 2.

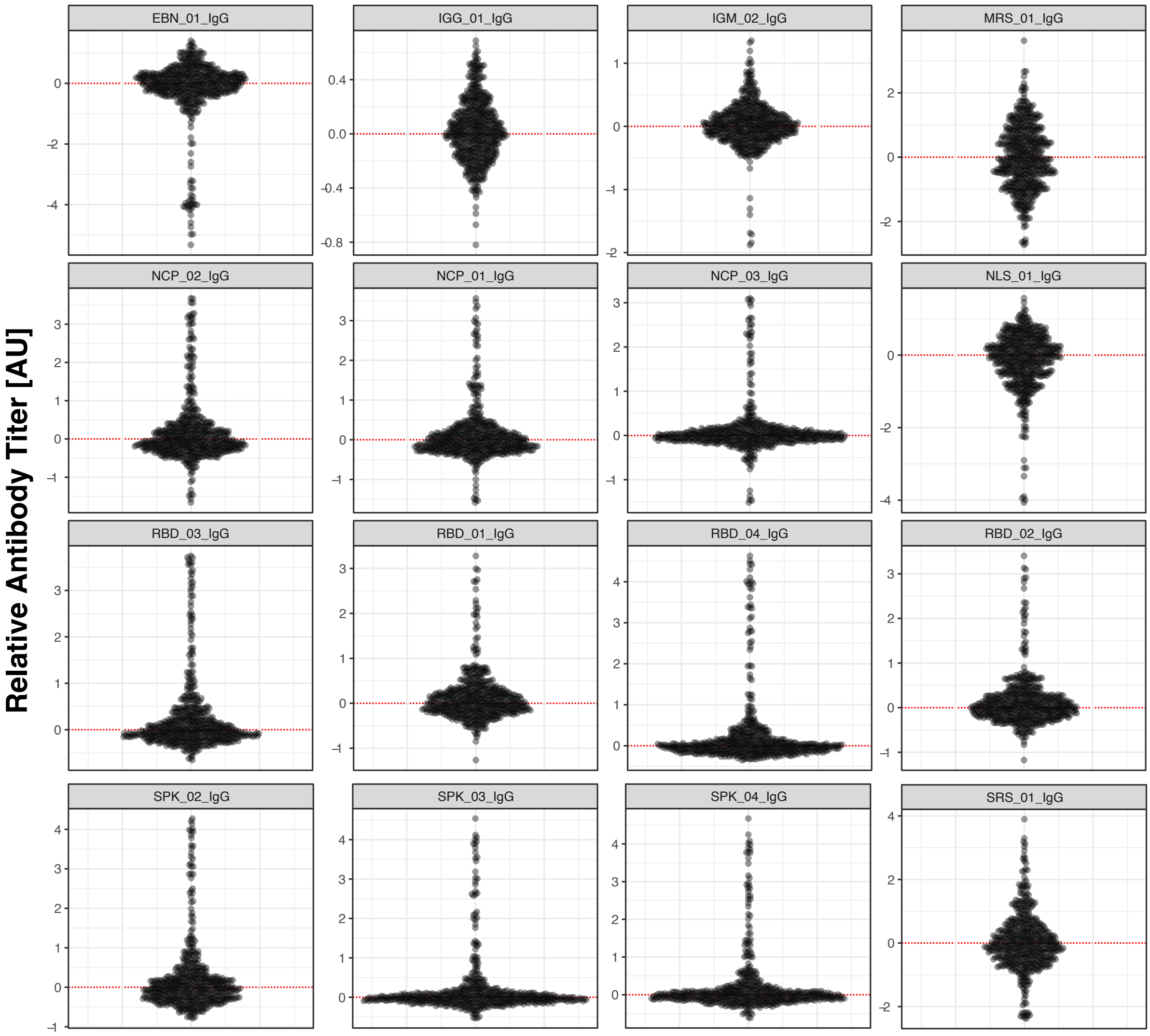

The relative IgG antibody titers detected in DBS are shown per antigen using the acronyms described in Table 1 of the Methods. The data represent the population, negative and positive controls samples. Source data are provided as a Source Data file.

Fig. S9D. IgM serology from study set 2.

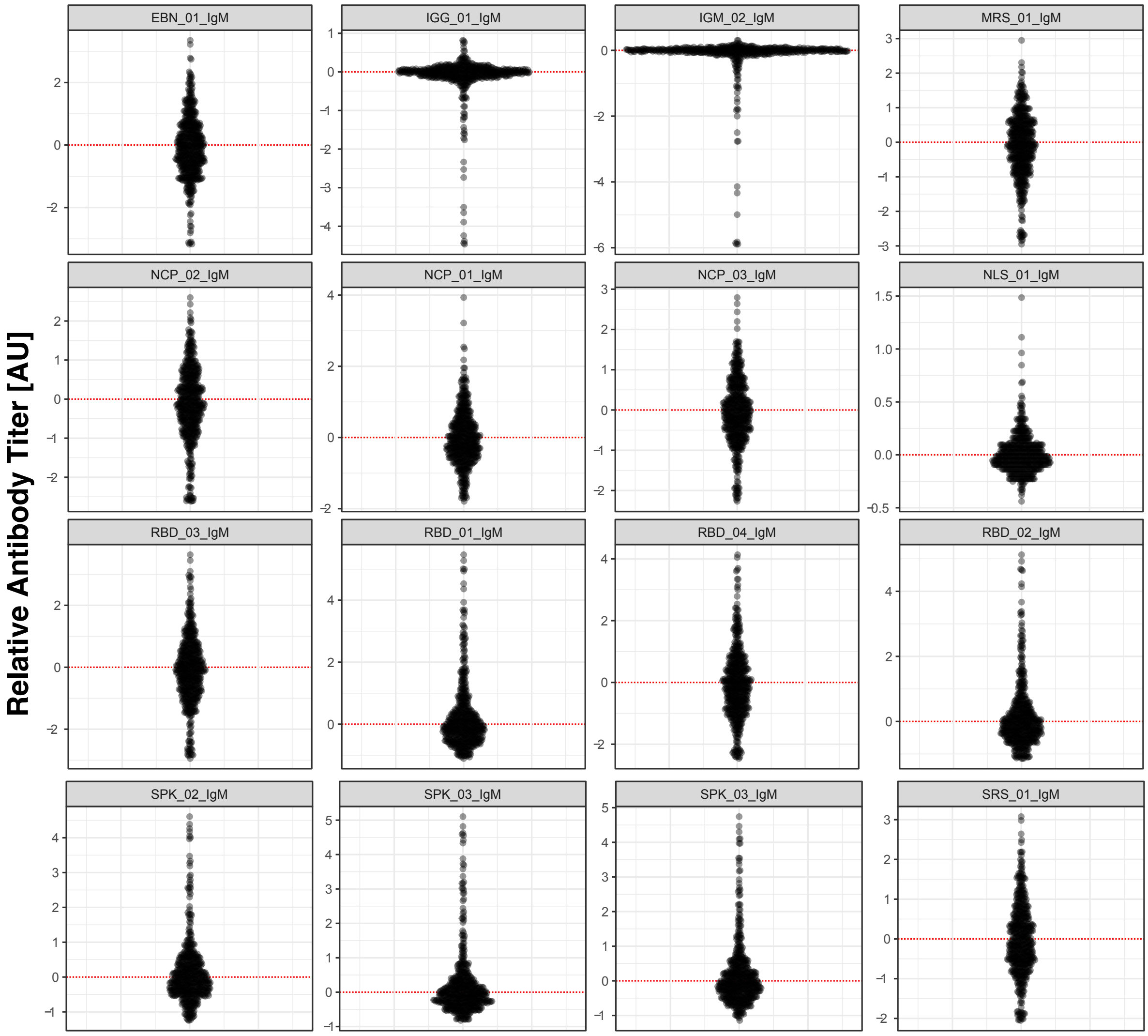

The relative IgM antibody titers detected in DBS are shown per antigen using the acronyms described in Table 1 of the Methods. The data represent the population, negative and positive controls samples. Source data are provided as a Source Data file.

Fig. S10A. PCA analyses of study set 1.

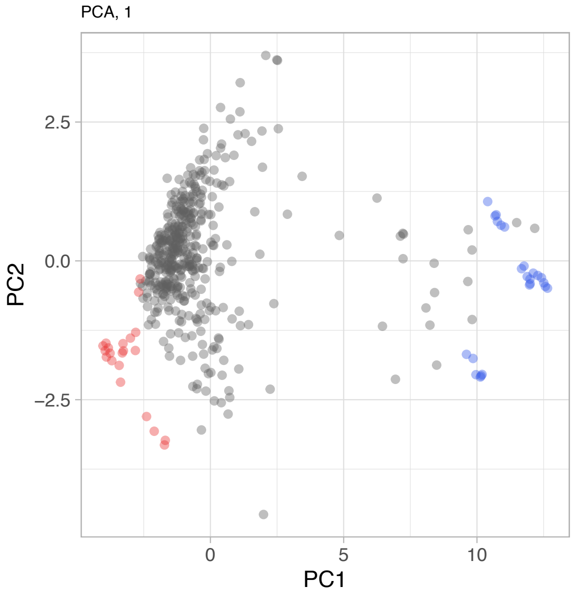

PCA was used to identify clusters of seropositivity by combining the IgG and IgM data. The colours indicate the samples from the population (grey), negative controls collected prior to 2020 (red) and PCR-confirmed positive controls (blue). The analysis was based on the S, RBD and N proteins: SPK_01, SPK_02, RBD_01, RBD_03, NCP_01. Source data are provided as a Source Data file.

Fig. S10B. PCA analyses of study set 2.

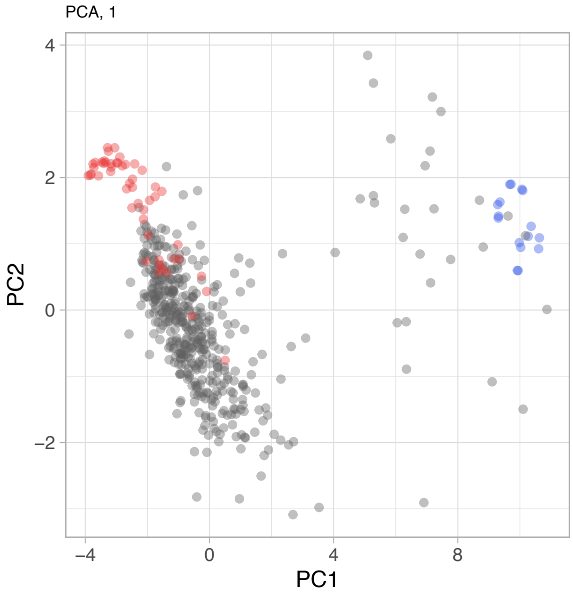

PCA was used to identify clusters of seropositivity by combining the IgG and IgM data. The colours indicate the samples from the population (grey), negative controls collected prior to 2020 (red) and PCR-confirmed positive controls (blue). The analysis was based on the S, RBD and N proteins: SPK_02, RBD_01, RBD_02, RBD_03, NCP_01. Source data are provided as a Source Data file.

Fig. S11A. IgG profiles of UMAP-selected seropositive and seronegative individuals.

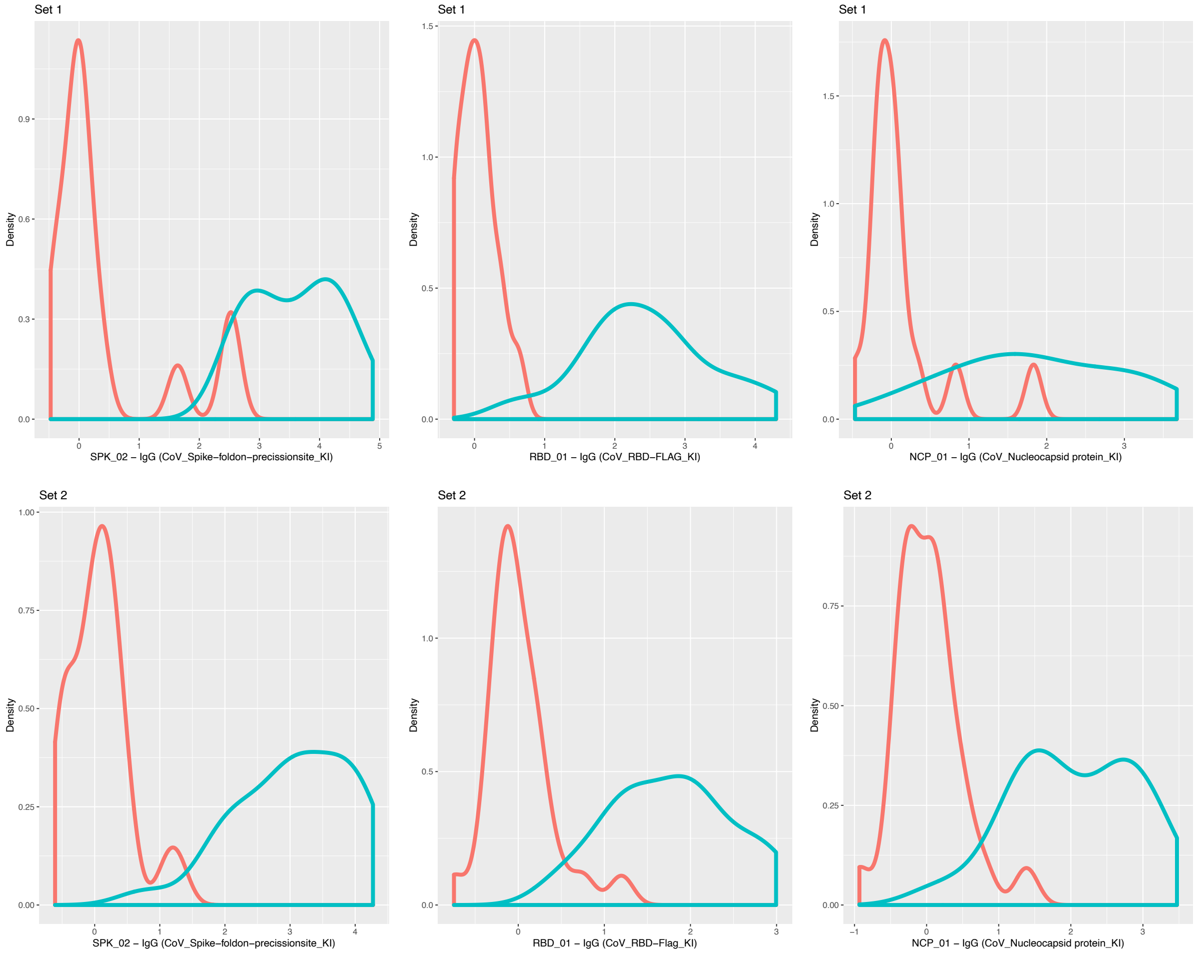

The density distributions are shown for the relative IgG titers (x-axis) in study set 1 (top row) and set 2 (bottom row) for S proteins (left panel), RBD (centre) and N proteins (right panel). The seropositive samples (petrol) were selected from the UMAP analysis and matched for sex, age and influenza-like symptom with seronegative samples (red). Source data are provided as a Source Data file.

Fig. S11B. IgM profiles of UMAP-selected seropositive and seronegative individuals.

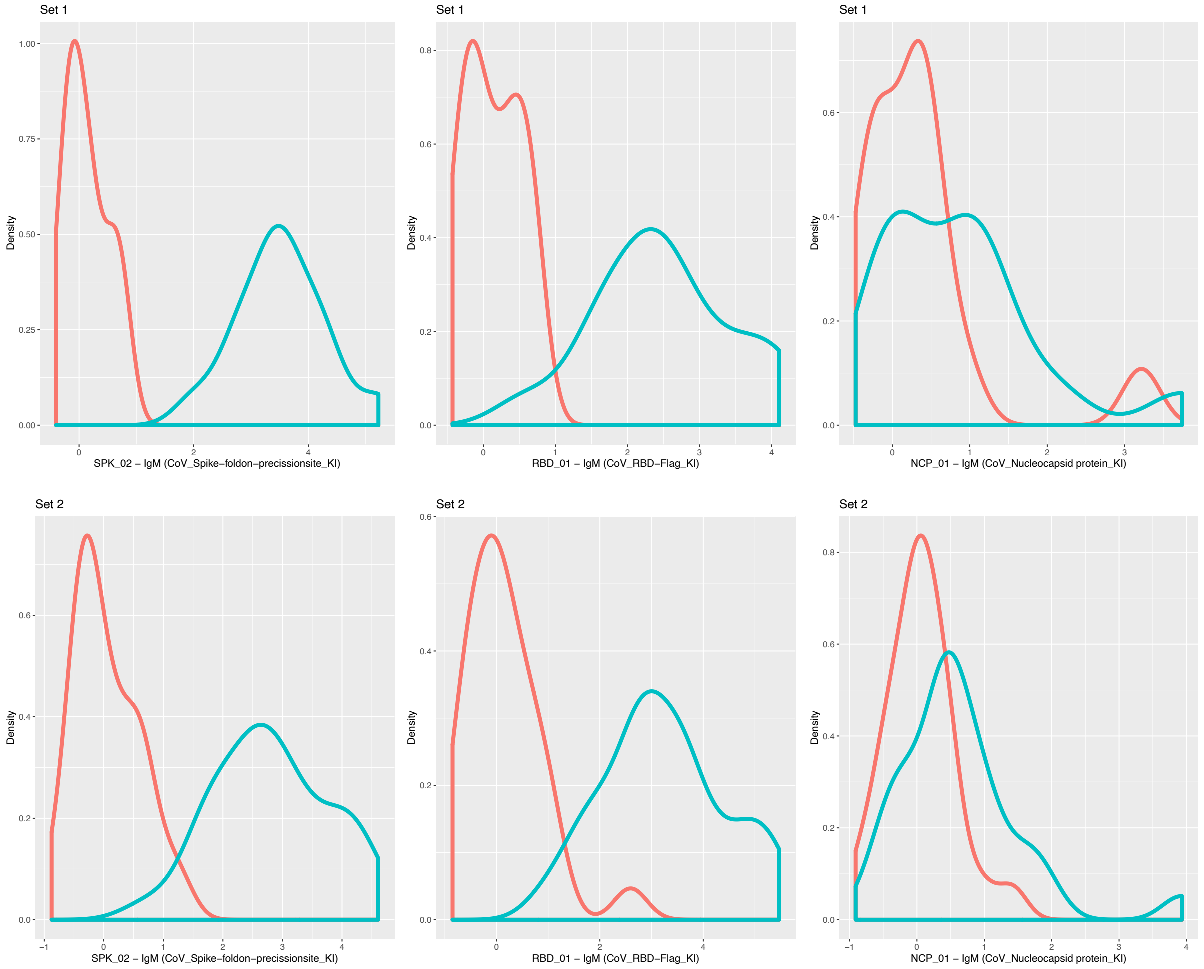

The density distributions are shown for the relative IgM titers (x-axis) in study set 1 (top row) and set 2 (bottom row) for S proteins (left panel), RBD (centre) and N proteins (right panel). The seropositive samples (petrol) were selected from the UMAP analysis and matched for sex, age and influenza-like symptom with seronegative samples (red). Source data are provided as a Source Data file.

Fig. S12A. Overlap in seropositive samples classified for IgG and IgM per study set.

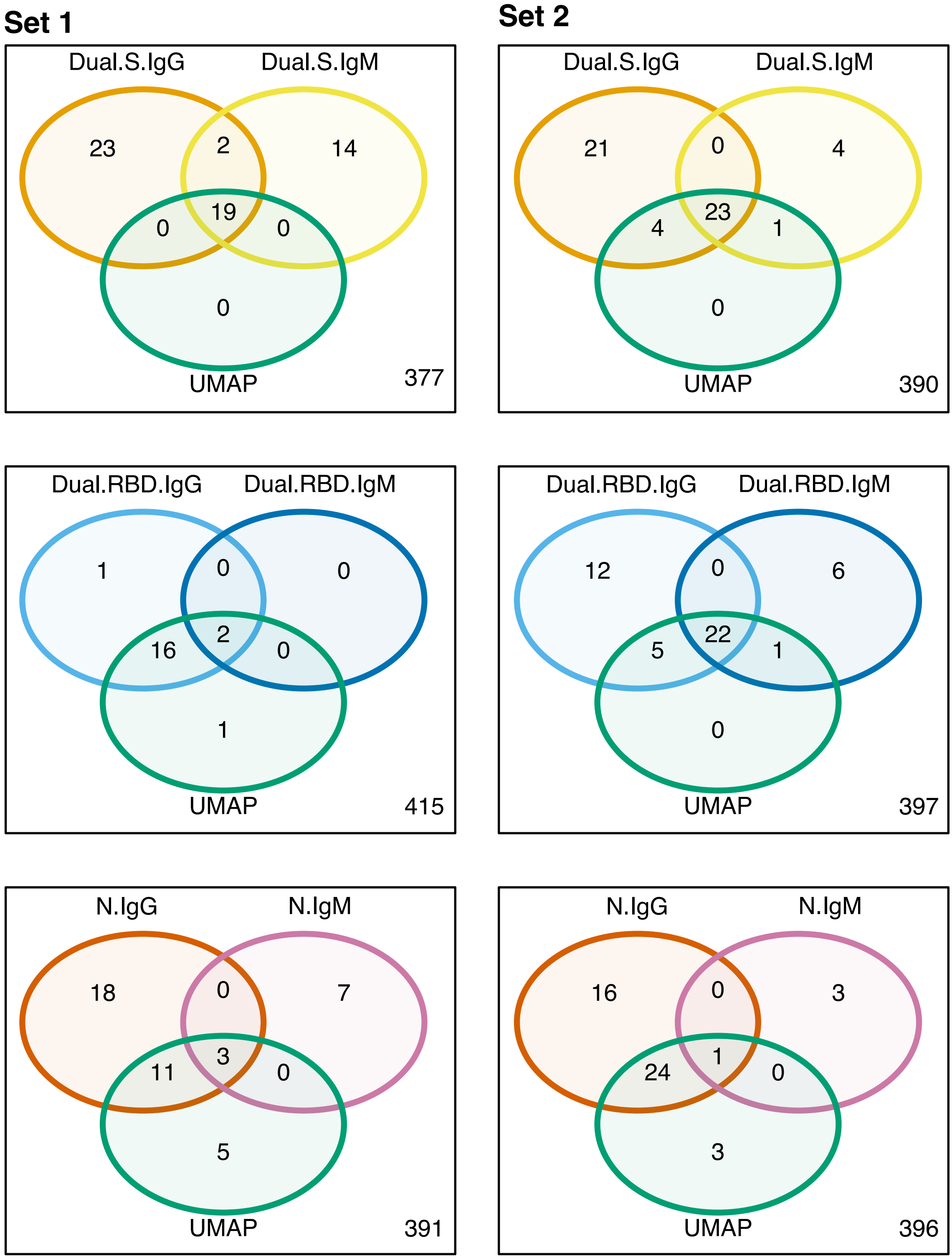

Venn diagrams showing the overlap in between seropositive samples obtained by S, RBD, and N proteins in relation to the UMAP method. The focus was to compare the classification of IgG+ and IgM+ samples in study set 1 (left) and set 2 (right). Source data are provided as a Source Data file.

Fig. S12B. Prevalence of IgG against SARS-CoV-2 proteins in study set 1.

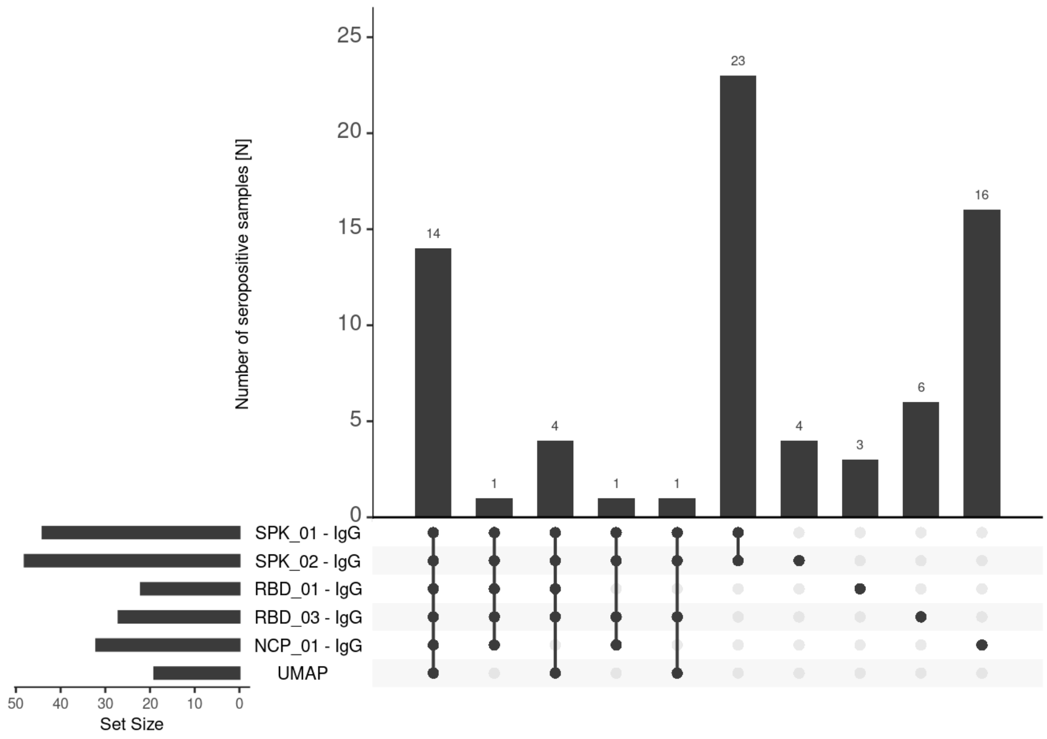

Overlap and number of samples classified as seropositive via the detection of IgG antibodies against different SARS-CoV-2 proteins and those of the UMAP cluster.

Fig. S12C. Prevalence of IgM against SARS-CoV-2 proteins in study set 1.

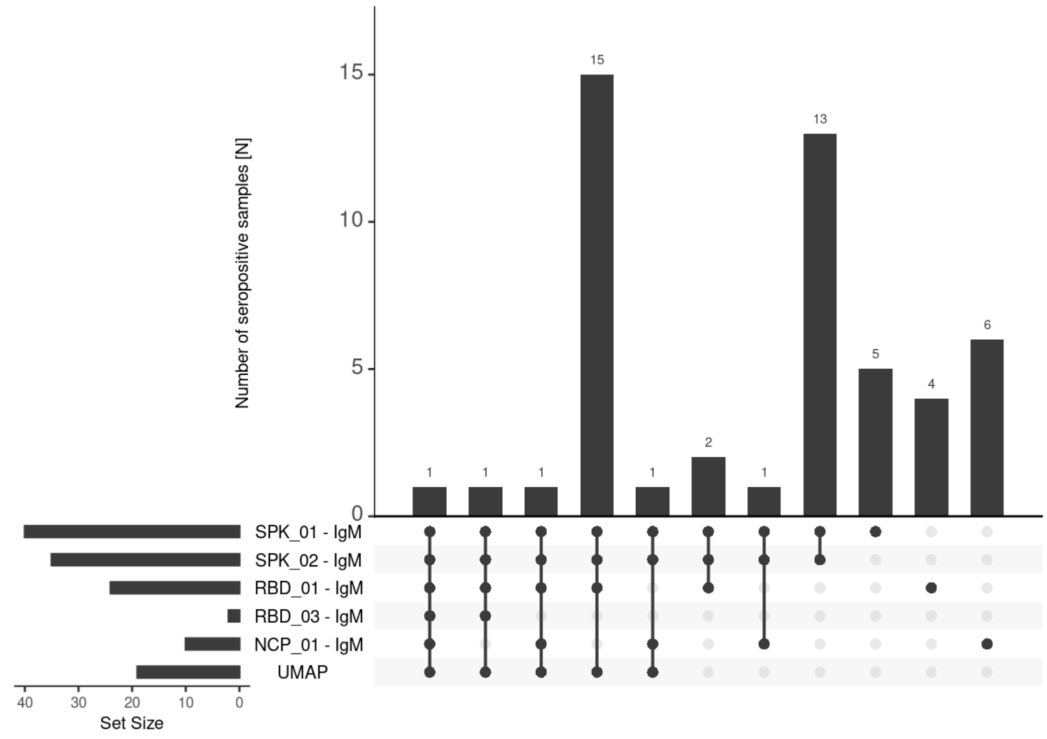

Overlap and number of samples classified as seropositive via the detection of IgM antibodies against different SARS-CoV-2 proteins and those of the UMAP cluster.

Fig. S12D Prevalence of IgG against SARS-CoV-2 proteins in study set 2.

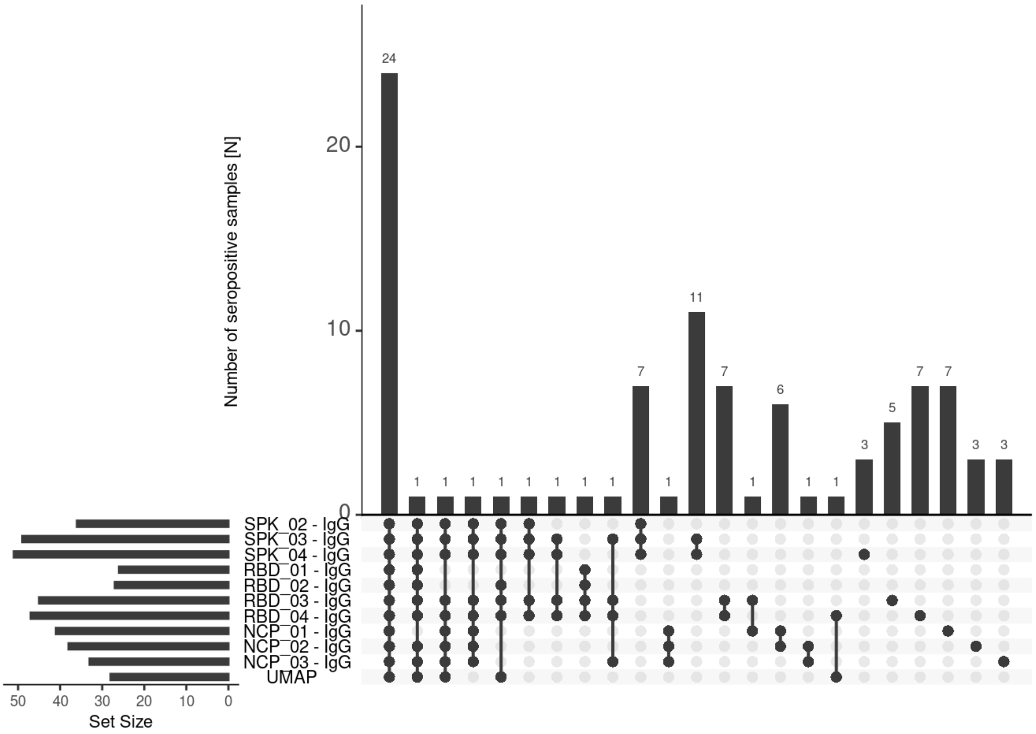

Overlap and number of samples classified as seropositive via the detection of IgG antibodies against different SARS-CoV-2 proteins and those of the UMAP cluster.

Fig. S12E Prevalence of IgM against SARS-CoV-2 proteins in study set 2.

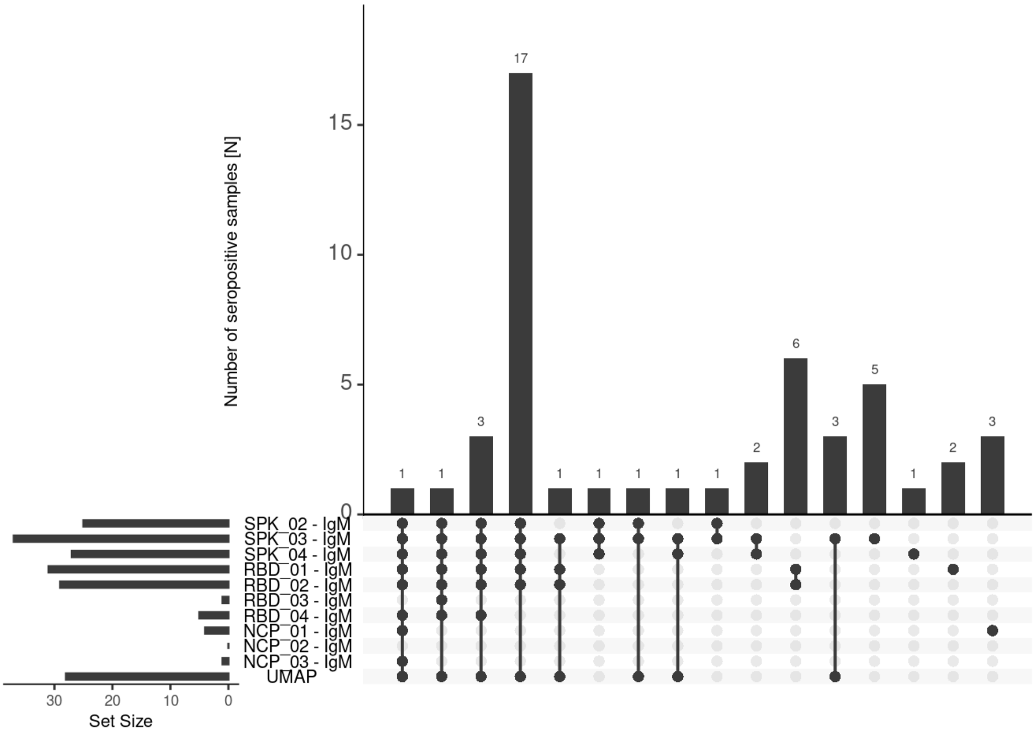

Overlap and number of samples classified as seropositive via the detection of IgM antibodies against different SARS-CoV-2 proteins and those of the UMAP cluster.

Supplementary Tables:

Table S1. Inter- and Intra-day variability.

Protein	Acronym	Intra-day CV			Inter-day CV
		Average	min	max	
**N**	NCP_01	11.5%	2.5%	19.1%	20.6%
	NCP_02	14.6%	7.2%	24.1%	14.7%
	NCP_03	13.8%	8.4%	20.4%	17.1%
**RBD**	RBD_01	13.6%	4.6%	25.3%	11.9%
	RBD_02	13.9%	4.6%	22.7%	16.7%
	RBD_03	14.8%	4.8%	26.9%	16.9%
	RBD_04	12.1%	5.3%	21.7%	22.1%
**S**	SPK_02	9.9%	4.9%	14.2%	21.1%
	SPK_03	11.5%	3.9%	22.0%	16.1%
	SPK_04	13.3%	4.6%	21.4%	21.7%

The stated coefficients of variation (CV) were determined on a unique blood samples eluted and processed in quadruplicates (intra-day) over four days (inter-day).

Table S2. Demographic characteristics of the pilot study.

	Study Set	Positive	Negative	P-value (OR)
	50 (100%)	24 (48%)	26 (52%)	
**Sex**				
Female	39 (78.0%)	20 (83.3%)	19 (73.1%)	0.50 (1.8)
Male	11 (22.0%)	4 (16.7%)	7 (26.9%)	
Other	0 (0%)	0 (0%)	0 (0%)	
Missing	0 (0%)	0 (0%)	0 (0%)	
**Age Groups**				
20-29	1 (2.0%)	0 (0%)	1 (3.8%)	0.67
30-39	12 (24.0%)	6 (25.0%)	6 (23.1%)	
40-49	12 (24.0%)	5 (20.8%)	7 (26.9%)	
50-59	15 (30.0%)	8 (33.3%)	7 (26.9%)	
60-69	8 (16.0%)	4 (16.7%)	4 (15.4%)	
70-74	2 (4.0%)	1 (4.2%)	1 (3.8%)	
Missing	0 (0%)	0 (0%)	0 (0%)	
**Influenza-like symptom**				
No	19 (38.0%)	2 (8.3%)	17 (65.4%)	< 0.001 (20.8)
Yes, mild	13 (26.0%)	8 (33.3%)	5 (19.2%)	
Yes, fever	16 (32.0%)	12 (50.0%)	4 (15.4%)	
Yes, severe	2 (4.0%)	2 (8.3%)	0 (0%)	
Missing	0 (0%)	0 (0%)	0 (0%)	
**Breathing symptoms***				
No	29 (58.0%)	10 (41.7%)	19 (73.1%)	0.04 (4.0)
Yes	21 (42%)	14 (58.3%)	7 (26.9%)	
Missing	0 (0%)	0 (0%)	0 (0%)	

Wilcoxon rank sum tests were used to determine the nominal p-values for the variables age and influenza-like symptoms. Fisher's exact tests were used for the binary variables sex and breathing. Both tests were two-sided. Odd ratios (OR) were calculated for the seropositive groups of females, those reporting no influenza-like symptoms and no issues related to breathing. The exact p-value reported as $p < 0.001$ was $p = 3.87e-05$.

*Defined as dry coughing or out of breath during at least a full week period the previous 3 months from the sampling date.

Table S3. Demographics of two study sets from random population sampling.

	Study Set 1	Study Set 2	Overall
**Participation**			
**Cards distributed**	1000	1000	2000
**Cards returned**	529 (48.2%)	568 (51.8%)	1097 (54.8%)*
**Cards approved**	435 (49.5%)	443 (50.5%)	878 (43.9%)*
**Sex**			
**Female**	239 (54.9%)	244 (55.1%)	483 (55.0%)
**Male**	170 (39.1%)	177 (40.0%)	347 (39.5%)
**Other**	1 (0.2%)	0 (0%)	1 (0.1%)
**Missing**	25 (5.7%)	22 (5.0%)	47 (5.4%)
**Age Groups**			
**20-29**	67 (15.4%)	68 (15.3%)	135 (15.4%)
**30-39**	77 (17.7%)	97 (21.9%)	174 (19.8%)
**40-49**	74 (17.0%)	91 (20.5%)	165 (18.8%)
**50-59**	87 (20.0%)	64 (14.4%)	151 (17.2%)
**60-69**	66 (15.2%)	64 (14.4%)	130 (14.8%)
**70-74**	39 (9.0%)	40 (9.0%)	79 (9.0%)
**Missing**	25 (5.7%)	19 (4.3%)	44 (5.0%)
**Influenza-like symptoms**			
**No**	221 (50.8%)	246 (55.5%)	467 (53.2%)
**Yes, mild**	122 (28.0%)	113 (25.5%)	235 (26.8%)
**Yes, fever**	58 (13.3%)	58 (13.1%)	116 (13.2%)
**Yes, severe**	9 (2.1%)	5 (1.1%)	14 (1.6%)
**Missing**	25 (5.7%)	21 (4.7%)	46 (5.2%)
**Breath symptoms**			
**No**	325 (74.7%)	315 (71.1%)	640 (72.9%)
**Yes**	85 (19.5%)	108 (24.4%)	193 (22.0%)
**Missing**	25 (5.7%)	20 (4.5%)	45 (5.1%)

* Related to total number of cards distributed

Table S4. Seroprevalence per antibody isotype and SARS-CoV-2 protein.

Protein	Antigen	Set	IgG				IgM			
			Prev.	95% CI	Pos.	Neg.	Prev.	95% CI	Pos.	Neg.
**N**	NCP_01	1	7.4%	4.9%- 9.8%	32	403	2.3%	0.9%-3.7%	10	425
**RBD**	RBD_01	1	5.1%	3.0%- 7.1%	22	413	5.5%	3.4%-7.7%	24	411
	RBD_02	1	6.2%	3.9%- 8.5%	27	408	0.5%	0.0%-1.1%	2	433
**S**	SPK_01	1	10.1%	7.3%-13.0%	44	391	9.2%	6.5%-11.9%	40	395
	SPK_02	1	11.1%	8.1%-14.0%	48	387	8.1%	5.5%-10.6%	35	400
**N**	NCP_01	2	9.3%	6.6%-12.0%	41	402	0.9%	0.0%-1.8%	4	439
	NCP_02	2	8.6%	6.0%-11.2%	38	405	0.0%	0.0%	0	443
	NCP_03	2	7.5%	5.0%-10.0%	33	410	0.2%	0.0%-0.7%	1	442
**RBD**	RBD_01	2	5.9%	3.7%-8.1%	26	417	7.0%	4.6%-9.4%	31	412
	RBD_02	2	6.1%	3.9%-8.3%	27	416	0.2%	0.0%-0.7%	1	442
	RBD_03	2	10.2%	7.3%-13.0%	45	398	6.6%	4.2%-8.9%	29	414
	RBD_04	2	10.6%	7.7%-13.5%	47	396	1.1%	0.1%-2.1%	5	438
**S**	SPK_02	2	8.1%	5.6%-10.7%	36	407	5.7%	3.5%-7.8%	25	418
	SPK_03	2	11.1%	8.2%-14.0%	49	394	8.4%	5.8%-10.9%	37	406
	SPK_04	2	11.5%	8.5%-14.5%	51	392	6.1%	3.9%- 8.3%	27	416

Prevalence levels were calculated on 6x SD in each study set. Numbers of samples above (Pos.) and below (Neg.) this cut-off are listed. Performance on sensitivity (Sens), specificity (Spec), were calculated using the included positive and negative controls. For set 1, there were 24 positive controls (4 unique plasma donors) and 45 negative controls (25 unique DBS blood donors). For set 2, there were 16 positive controls (2 unique DBS blood donors) and 50 negative controls (50 unique DBS blood donors).

Table S5. Demographics of subjects in UMAP group.

Selection	UMAP		
	Positive	Negative	P-value (OR)
**Samples**			
**Groups**	47 (5.4%)	831 (94.6%)	
**Sex**			
**Female**	28 (59.6%)	455 (54.8%)	0.8 (0.94)
**Male**	19 (40.4%)	328 (39.5%)	
**Other**	0 (0%)	1 (0.1%)	
**Missing**	0 (0%)	47 (5.7%)	
**Age group (years)**			
**20-29**	15 (31.9%)	120 (14.4%)	0.006 (N/A)
**30-39**	10 (21.3%)	164 (19.7%)	
**40-49**	8 (17.0%)	157 (18.9%)	
**50-59**	6 (12.8%)	145 (17.4%)	
**60-69**	7 (14.9%)	123 (14.8%)	
**70-74**	1 (2.1%)	78 (9.4%)	
**Missing**	0 (0%)	44 (5.3%)	
**Influenza-like symptoms**			
**No**	8 (17.0%)	459 (55.2%)	< 0.001 (6.67)
**Yes, mild**	15 (31.9%)	220 (26.5%)	
**Yes, fever**	20 (42.6%)	96 (11.6%)	
**Yes, severe**	4 (8.5%)	10 (1.2%)	
**Missing**	0 (0%)	46 (5.5%)	
**Breathing symptoms**			
**No**	28 (59.6%)	612 (73.6%)	0.007 (2.38)
**Yes**	19 (40.4%)	174 (20.9%)	
**Missing**	0 (0%)	45 (5.4%)	

Wilcoxon rank sum tests were used to determine the nominal p-values for the variables age and influenza-like symptoms. Fisher's exact tests were used for the binary variables sex and breathing. Both tests were two-sided. Odd ratios (OR) were calculated for the seropositive groups of females, those reporting no influenza-like symptoms and no issues related to breathing. The exact p-value reported as $p < 0.001$ was $p = 2.03e-11$.

Table S6. Demographics of subjects dual-S group (including all samples).

Samples	IgG positive by dual-S		
	Positive	Negative	P-value (OR)
**Samples**			
**Groups**	92 (10.5%)	786 (89.5%)	
**Sex**			
**Female**	54 (58.7%)	429 (54.6%)	0.9 (1.05)
**Male**	37 (40.2%)	310 (39.4%)	
**Other**	0 (0%)	1 (0.1%)	
**Missing**	1 (1.1%)	46 (5.9%)	
**Age group (years)**			
**20-29**	24 (26.1%)	111 (14.1%)	0.11 (N/A)
**30-39**	14 (15.2%)	160 (20.4%)	
**40-49**	16 (17.4%)	149 (19.0%)	
**50-59**	17 (18.5%)	134 (17.0%)	
**60-69**	16 (17.4%)	114 (14.5%)	
**70-74**	4 (4.3%)	75 (9.5%)	
**Missing**	1 (1.1%)	43 (5.5%)	
**Influenza-like symptom**			
**No**	33 (35.9%)	434 (55.2%)	< 0.001 (2.50)
**Yes, mild**	27 (29.3%)	208 (26.5%)	
**Yes, fever**	27 (29.3%)	89 (11.3%)	
**Yes, severe**	4 (4.3%)	10 (1.3%)	
**Missing**	1 (1.1%)	45 (5.7%)	
**Breathing difficulty**			
**No**	60 (65.2%)	580 (73.8%)	0.012 (1.85)
**Yes**	31 (33.7%)	162 (20.6%)	
**Missing**	1 (1.1%)	44 (5.6%)	

Wilcoxon rank sum tests were used to determine the nominal p-values for the variables age and influenza-like symptoms. Fisher's exact tests were used for the binary variables sex and breathing. Both tests were two-sided. Odd ratios (OR) were calculated for the seropositive groups of females, those reporting no influenza-like symptoms and no issues related to breathing. The exact p-value reported as $p < 0.001$ was $p = 1.01e-06$.

Table S7. Comparisons of dual-S and UMAP group.

**Samples**			
	Dual-S-UMAP*	UMAP	P-value (OR)
**Samples**			
**Groups**	46	47	
**Sex**			
**Female**	27 (58.7%)	28 (59.6%)	1 (1.02)
**Male**	18 (39.1%)	19 (40.4%)	
**Other**	1 (2.2%)	0 (0%)	
**Missing**	1 (2.2%)	0 (0%)	
**Age group (years)**			
**20-29**	9 (19.6%)	15 (31.9%)	0.03 (N/A)
**30-39**	4 (8.7%)	10 (21.3%)	
**40-49**	9 (19.6%)	8 (17.0%)	
**50-59**	11 (23.9%)	6 (12.8%)	
**60-69**	9 (19.6%)	7 (14.9%)	
**70-74**	3 (6.5%)	1 (2.1%)	
**Missing**	1 (2.2%)	0 (0%)	
**Influenza-like symptoms**			
**No**	25 (54.3%)	8 (17.0%)	< 0.001 (6.09)
**Yes, mild**	13 (28.3%)	15 (31.9%)	
**Yes, fever**	7 (15.2%)	20 (42.6%)	
**Yes, severe**	0 (0%)	4 (8.5%)	
**Missing**	1 (2.2%)	0 (0%)	
**Breathing symptoms**			
**No**	33 (71.7%)	28 (59.6%)	0.19 (1.87)
**Yes**	12 (26.1%)	19 (40.4%)	
**Missing**	1 (2.2%)	0 (0%)	

Wilcoxon rank sum tests were used to determine the nominal p-values for the variables age and influenza-like symptoms. Fisher's exact tests were used for the binary variables sex and breathing. Both tests were two-sided. Odd ratios (OR) were calculated for the seropositive groups of females, those reporting no influenza-like symptoms and no issues related to breathing. The exact p-value reported as $p < 0.001$ was $p = 1.58e-05$.

* Excludes 45 samples from the dual-S group that were common with UMAP seropositive group.
