# Supplementary material for: Multianalyte serology in home-sampled blood enables an unbiased assessment of the immune response against SARS-CoV-2
Source: Nat Commun. 2021 Jun 17;12:3695. doi: 10.1038/s41467-021-23893-4 (PMC8211676; doi:10.1038/s41467-021-23893-4)
Supplement: Supplementary file 3 — Description of Additional Supplementary Files [file 41467_2021_23893_MOESM3_ESM.pdf]

Description of Additional Supplementary Files

File Name: Supplementary Data 1
Description: The file contains tables concerning the performance of the antigens in the different assays, in sheets named Prevalence per Antigen, Combinatorial prevalence, Paired antigens pilot test, Paired samples pilot test)
