# Supplementary material for: Multianalyte serology in home-sampled blood enables an unbiased assessment of the immune response against SARS-CoV-2
Source: Nat Commun. 2021 Jun 17;12:3695. doi: 10.1038/s41467-021-23893-4 (PMC8211676; doi:10.1038/s41467-021-23893-4)
Supplement: Supplementary file 5 — Reporting Summary [file 41467_2021_23893_MOESM5_ESM.pdf]

nature research

Corresponding author(s): Niclas Roxhed, Jochen Schwenk

Last updated by author(s): May 4, 2021

Reporting Summary

Nature Research wishes to improve the reproducibility of the work that we publish. This form provides structure for consistency and transparency in reporting. For further information on Nature Research policies, see our Editorial Policies and the Editorial Policy Checklist.

Statistics

For all statistical analyses, confirm that the following items are present in the figure legend, table legend, main text, or Methods section.

n/a	Confirmed	
☐	☒	The exact sample size (n) for each experimental group/condition, given as a discrete number and unit of measurement
☐	☒	A statement on whether measurements were taken from distinct samples or whether the same sample was measured repeatedly
☐	☒	The statistical test(s) used AND whether they are one- or two-sided<br>*Only common tests should be described solely by name; describe more complex techniques in the Methods section.*
☐	☒	A description of all covariates tested
☐	☒	A description of any assumptions or corrections, such as tests of normality and adjustment for multiple comparisons
☐	☒	A full description of the statistical parameters including central tendency (e.g. means) or other basic estimates (e.g. regression coefficient) AND variation (e.g. standard deviation) or associated estimates of uncertainty (e.g. confidence intervals)
☐	☒	For null hypothesis testing, the test statistic (e.g. F, t, r) with confidence intervals, effect sizes, degrees of freedom and P value noted<br>*Give P values as exact values whenever suitable.*
☒	☐	For Bayesian analysis, information on the choice of priors and Markov chain Monte Carlo settings
☒	☐	For hierarchical and complex designs, identification of the appropriate level for tests and full reporting of outcomes
☒	☐	Estimates of effect sizes (e.g. Cohen's d, Pearson's r), indicating how they were calculated

Our web collection on statistics for biologists contains articles on many of the points above.

Software and code

Policy information about availability of computer code

Data collection	Data was collected on Luminex FlexMap 3D instruments operated by the xPONENT software version 4.3
Data analysis	Data processing and analyses were performed in v.3.6.0 of R or Julia. Analysis scripts are available at https://github.com/Schwenk-Lab/covid19-serology

For manuscripts utilizing custom algorithms or software that are central to the research but not yet described in published literature, software must be made available to editors and reviewers. We strongly encourage code deposition in a community repository (e.g. GitHub). See the Nature Research guidelines for submitting code & software for further information.

Data

Policy information about availability of data

All manuscripts must include a data availability statement. This statement should provide the following information, where applicable:

- Accession codes, unique identifiers, or web links for publicly available datasets
- A list of figures that have associated raw data
- A description of any restrictions on data availability

Normalized and anonymized serology data of the population studies can be made available for validation purposes and upon reasonable request to the corresponding authors.

Field-specific reporting

Please select the one below that is the best fit for your research. If you are not sure, read the appropriate sections before making your selection.

☒ Life sciences ☐ Behavioural & social sciences ☐ Ecological, evolutionary & environmental sciences

For a reference copy of the document with all sections, see nature.com/documents/nr-reporting-summary-flat.pdf

Life sciences study design

All studies must disclose on these points even when the disclosure is negative.

Sample size	No sample size calculation was performed. We aimed at collecting 2000 samples in sets of 1000 and assumed the willingness to participate and success rate to collect samples to be the limiting factor. Per set, we aimed at analysing 300 samples (30% of all). We analysed 878 samples in the end (44% of all).
Data exclusions	There were no data exclusion criteria. Only samples and questionnaire data from donors providing at least one disc filled with 10 µl of blood were then analyzed.
Replication	The analysis of the population samples were conducted once. Replicated analysis of samples (see Table S1) revealed inter-assays CVs of 12%-22%.
Randomization	The study was observational and randomization was not implemented.
Blinding	The SARS-CoV-2 serostatus of all samples was unknown until the samples were analyzed. Demographic information was available prior to the analysis.

Reporting for specific materials, systems and methods

We require information from authors about some types of materials, experimental systems and methods used in many studies. Here, indicate whether each material, system or method listed is relevant to your study. If you are not sure if a list item applies to your research, read the appropriate section before selecting a response.

Materials & experimental systems

n/a	Involved in the study	
☐	☒	Antibodies
☒	☐	Eukaryotic cell lines
☒	☐	Palaeontology and archaeology
☒	☐	Animals and other organisms
☐	☒	Human research participants
☒	☐	Clinical data
☒	☐	Dual use research of concern

Methods

n/a	Involved in the study	
☒	☐	ChIP-seq
☒	☐	Flow cytometry
☒	☐	MRI-based neuroimaging

Antibodies

Antibodies used	1) anti-human IgG-R-PE (H10104, Lot# 457 2079224, Invitrogen),<br>2) anti-human IgM-R-PE (#109-116-129, Lot# 137465, Jackson Immunoresearch),<br>3) Anti-human IgG (#309-005-082, Lot# 132463, Jackson ImmunoResearch),<br>4) anti-human IgM (#109-005-129, Lot# 147777, Jackson 445 ImmunoResearch),<br>5) anti-human IgA (#GA-80A, Lot# 0017, Immune Systems Ltd)
Validation	1) https://www.thermofisher.com/antibody/product/Goat-anti-Human-IgG-Fc-Secondary-Antibody-Polyclonal/H10104<br>2) https://www.jacksonimmuno.com/catalog/products/109-116-129<br>3) https://www.jacksonimmuno.com/catalog/products/309-005-082<br>4) https://www.jacksonimmuno.com/catalog/products/109-005-129<br>5) https://www.immunesystems.co.uk/catalog/product_info.php?products_id=575

Human research participants

Policy information about studies involving human research participants

Population characteristics	Demographics available from the population and pilot study are given in Tables S3 and S4 and summary information of PCR confirmed COVID19 positive donors have now been added, when data was available.

Recruitment	Participants randomly selected from public address registers were invited by regular mail. The sampling letters contained the collection tools and a return envelope. Only samples returned within the study time frame and that DBS containing 10 µl of blood were analyzed. No parameters other than metropolitan Stockholm, age and sex were used as inclusion criteria. Possible sources of bias were the availability and willingness to participate, as well as the capability to collected self-sampled blood. These possible sources of bias may have influenced the total number of participants and how many were represented in the different age and sex groups.
Ethics oversight	The study was approved by the regional ethical board (EPN Stockholm, Dnr 2015/867-31/1) and the Swedish Ethical Review Authority (EPM, Dnr 2020-01500). Use of biobanked controls samples was approved by the Swedish Ethical Review Authority (Dnr 2020-02483).

Note that full information on the approval of the study protocol must also be provided in the manuscript.
